# Microbiota Modulates Cardiac Transcriptional Responses to Intermittent Hypoxia and Hypercapnia

**DOI:** 10.3389/fphys.2021.680275

**Published:** 2021-06-25

**Authors:** Dan Zhou, Jin Xue, Yukiko Miyamoto, Orit Poulsen, Lars Eckmann, Gabriel G. Haddad

**Affiliations:** ^1^Division of Respiratory Medicine, Department of Pediatrics, University of California, San Diego, La Jolla, CA, United States; ^2^Department of Medicine, University of California, San Diego, La Jolla, CA, United States; ^3^Department of Neurosciences, University of California, San Diego, La Jolla, CA, United States; ^4^Rady Children’s Hospital-San Diego, San Diego, CA, United States

**Keywords:** gnotobiotics, transcriptome, intermittent hypoxia and hypercapnia, heart, mice

## Abstract

The microbiota plays a critical role in regulating organismal health and response to environmental stresses. Intermittent hypoxia and hypercapnia, a condition that represents the main hallmark of obstructive sleep apnea in humans, is known to induce significant alterations in the gut microbiome and metabolism, and promotes the progression of atherosclerosis in mouse models. To further understand the role of the microbiome in the cardiovascular response to intermittent hypoxia and hypercapnia, we developed a new rodent cage system that allows exposure of mice to controlled levels of O_2_ and CO_2_ under gnotobiotic conditions. Using this experimental setup, we determined the impact of the microbiome on the transcriptional response to intermittent hypoxia and hypercapnia in the left ventricle of the mouse heart. We identified significant changes in gene expression in both conventionally reared and germ-free mice. Following intermittent hypoxia and hypercapnia exposure, we detected 192 significant changes in conventionally reared mice (96 upregulated and 96 downregulated) and 161 significant changes (70 upregulated and 91 downregulated) in germ-free mice. Only 19 of these differentially expressed transcripts (∼10%) were common to conventionally reared and germ-free mice. Such distinct transcriptional responses imply that the host microbiota plays an important role in regulating the host transcriptional response to intermittent hypoxia and hypercapnia in the mouse heart.

## Introduction

Obstructive sleep apnea (OSA) is the most common type of sleep disorder in humans, and OSA is very prevalent in adults and children. It is characterized by repeated episodes of complete or partial obstructions of the upper airways during sleep, which is associated with a reduction in blood oxygen saturation and an increase in blood CO_2_. It has been demonstrated by experimental and clinical studies that OSA may lead to a number of health consequences including cardiovascular diseases ranging from cardiometabolic disorders and arrhythmogenesis to heart failure (Al Lawati et al., 2009; Baguet et al., 2012; Levy et al., 2013; Drager et al., 2015; Lyons and Bradley, 2015; Hoyos et al., 2017; May et al., 2017).

In addition, evidence from both animal and human studies supports the notion that the gut microbiota plays a critical role in the onset and progression of various cardiovascular diseases (Karbach et al., 2016; Koch et al., 2017; Weis, 2018). Previous studies by others and us have shown that intermittent hypoxia with/without hypercapnia alters the gut microbiome (Moreno-Indias et al., 2015, 2016; Lucking et al., 2018; Tripathi et al., 2018, 2019). In turn, such alterations may induce developmental defects and functional dysregulations. For example, the human oral and intestinal microbiota has been recognized as a significant determinant of cardiovascular disease risk (Ordovas and Mooser, 2006), and suppression of the gut microbiome ameliorates age-dependent arterial dysfunction (Brunt et al., 2019). Recent studies also revealed a potential contribution of gut microbes to certain human cardiometabolic diseases (Vinje et al., 2014) and heart failure (Sandek et al., 2007; Nagatomo and Tang, 2015; Nemet et al., 2020).

Besides impacting cellular signaling, the microbiome has been demonstrated to modify the epigenetic landscape of the host genome, which may influence cellular differentiation and proliferation, leading to new phenotypic traits throughout the age spectrum (Takahashi et al., 2011; Pevsner-Fischer et al., 2017; Qin and Wade, 2018). For example, several studies have demonstrated a direct link between the gut microbiome and host epigenetic states in the context of inflammatory bowel disease, obesity, cancer, and behavioral abnormalities (Kellermayer et al., 2010; Manichanh et al., 2012; Dejea et al., 2014; Paul et al., 2015; Farhana et al., 2018; Sfanos et al., 2018; Chu et al., 2019).

Since gut microbiota plays an important role in regulating host gene expression (Patrignani et al., 2014; Chu et al., 2019; Richards et al., 2019; Weger et al., 2019), in the current study, we tested the hypothesis that the microbiome regulates the transcriptional response to intermittent hypoxia and hypercapnia (IHH) exposure in the heart. Using a newly developed experimental setup to impose IHH under gnotobiotic conditions, we performed 150-bp paired-end RNA-seq and utilized multiple bioinformatic approaches to analyze the transcriptional alterations following IHH treatment in the left ventricle (LV) of conventionally reared (CONV) and germ-free (GF) C57BL/6 mice. In addition, we compared our results with those reported for other organs in male GF mice of similar genetic background and age (Weger et al., 2019) (dataset GSE77221). We found significant and distinct differences in transcriptional responses between CONV and GF mice, demonstrating that, indeed, the microbiome plays an important role in regulating IHH response in the heart.

## Materials and Methods

### Experimental Animals

GF and CONV C57BL/6 mice that were 8–12 weeks old were used for this experiment. GF status was confirmed weekly by standard microbiological cultures of fecal extracts, using thioglycollate broth and potato dextrose broth for facultative anaerobes including fungi, and cooked meat broth, heart infusion broth, and Columbia blood agar for obligate anaerobes (Moody et al., 2019). All animal protocols were approved by the Animal Care Committee of the University of California, San Diego and followed the Guide for the Care and Use of Laboratory Animals of the National Institutes of Health.

### IHH Treatment

**FIGURE 1:**
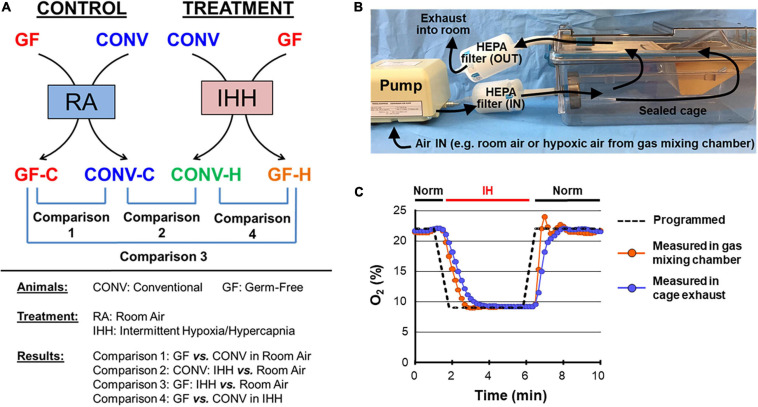
Experiment design and instrumentation. **(A)** Conventional (CONV) and germ-free (GF) mice were treated with intermittent hypoxia/hypercapnia (IHH) or room air (RA) to determine the impacts of microbiome on heart transcriptome under normoxia and transcriptional response to IHH stress. **(B)** A tightly sealed cage system was developed to allow control of the cage atmosphere under gnotobiotic conditions by ventilating cages with a single, HEPA-filtered gas. The gnotobiotic chamber for treatment was supplied with IHH gas; room air was supplied to the control chamber. **(C)** A representative recording of a test run of the experimental setup with intermittent hypoxia (IH). The gas environment inside the cage was controlled by placing the pump inlet into a gas mixing chamber. IH was induced in the cage by obtaining gas from the gas mixing chamber with a programmed cycle of 5 min of 8% O_2_ followed by 5 min of room air (21% O_2_) (dashed black line). O_2_ levels were measured directly in the mixing chamber (red symbols) or in the exhaust gas of the ventilated cage (blue symbols). IH, intermittent hypoxia; Norm, normoxia.

All mice were housed in specialized cages for the 2-week treatments (IHH or normoxia; GF or CONV) (Figure 1). These specialized cages were developed by us in the Gnotobiotic Animal Facility at the University of California, San Diego. Each sealed single cage (Sealed Positive Pressure System, Allentown, NJ, United States) was ventilated by a single, HEPA (high-efficiency particulate air)-filtered gas supply pump. The cages were opened and closed for stocking and servicing inside a Class IIa laminar-flow biosafety cabinet. The system allows complete control of gas inflow and outflow, as has been confirmed by measurements of exhaust gases using an O_2_/CO_2_ analyzer (Quantek Instruments, Grafton, MA, United States). The IHH condition was maintained in a computer-controlled atmosphere chamber system (OxyCycler, BioSpherix, Redfield, NY, United States) as previously described (Fan et al., 2005; Zhou et al., 2008). IHH exposure was introduced to the mice in short periods of ∼4 min of synchronized reduction in (O_2_) (from 21 to 8%) and increase in (CO_2_) (from ∼0.5 to 8%) separated by alternating ∼4-min periods of normoxic room air [(O_2_) = 21% and (CO_2_) ≈ 0.5%] with 2 ramp intervals (∼1 min each) for a total of 10 IHH events per hour for 10 h per day during the light cycle. Controls received a flow of room air throughout the entire experimental period. Prior works have shown that the oxygen saturation level (SaO_2_) ranged from 60 to 80% when mice were subjected to 5–10% hypoxic treatments (Sforza and Roche, 2016; Xue et al., 2017).

### RNA Sequencing and Data Analysis

At the end of the IHH exposure, mice were sacrificed, and the heart was harvested and separated into the right and left ventricle under sterile conditions and then placed into separate tubes containing RNAlater Stabilization Solution (Thermo Fisher Scientific, Ashville, NC, United States). RNA from the LV was extracted using the RNeasy Mini Kit (QIAGEN, Hilden, Germany) and sent to Novogene (Chula Vista, CA, United States) for QC and sequencing (*n* = 3/group). A 150 nt paired-end sequencing on the Illumina HiSeq 4000 platform (Illumina, San Diego, CA, United States) was performed. Rigorous quality controls of the paired-end reads were assessed using FastQC tools, and the adapter sequences and low-quality bases were trimmed using Cutadapt (Didion et al., 2017). The resulting reads were processed using the Illumina BaseSpace software package using the STAR aligner (Dobin et al., 2013) to align reads against the *Mus musculus* UCSC mm9 reference genome. Differential expression was calculated using DESeq 2 with default settings (Anders and Huber, 2010), and differentially expressed genes (DEGs) were identified with a threshold of |Fold-Change| > 1.5 and adjusted *p*-value < 0.05. Bioinformatic data mining of the differentially expressed transcripts was carried out with the tools provided by the GWAS Catalog^1^ (Buniello et al., 2019), the Database for Annotation, Visualization, and Integrated Discovery (DAVID)^2^ (Dennis et al., 2003; Huang et al., 2007), the Ingenuity Pathway Analysis (IPA) package (QIAGEN, Redwood City, CA, United States), as well as KEGG^3^ (Ogata et al., 1999; Kanehisa and Goto, 2000) and the Reactome^4^ (Fabregat et al., 2017). Multiple testing correction was applied, and the FDR *q*-values were estimated to correct the *p*-values for the multiple testing issue.

### Data Availability

The paired-end RNA-seq data of *n* = 12 samples are available at https://www.ncbi.nlm.nih.gov/bioproject/692551 (BioProject ID: PRJNA692551).

## Results and Discussion

### Experimental Design

The microbiome, particularly in the gut, plays an important role in maintaining systemic homeostasis and contributes to a number of human diseases [for selected reviews, see Lynch and Pedersen (2016), Gilbert et al. (2018), Dominguez-Bello et al. (2019), Harikrishnan (2019), Helmink et al. (2019), Team (2019)]. However, its potential involvement in regulating many physiologic and pathophysiologic responses under stressful conditions remains to be explored. In the current study, we used a prospective case-control study design to determine the impact of the microbiome on cardiac transcriptional responses to IHH in CONV and GF mice (Figure 1A). To maintain GF animals under controlled gas conditions, we developed a custom cage system with minimal dead space and programmable HEPA-filtered gas supply (Figure 1B). Analysis of the exhaust gases of the cage system revealed rapid (<20 s) equilibration of the cage environment to match the input gas mixture (Figure 1C). GF mice could be kept in the system for extended periods (>2 weeks) without compromising their GF state, demonstrating that the newly developed system was well suited to conduct short- and long-term experiments of constant or intermittent hypoxia with or without hypercapnia under gnotobiotic conditions.

GF and CONV mice of the same strain, sex, and age (kept in the same cage system as the GF mice) were either exposed to the IHH condition for 2 weeks to mimic sleep apnea in humans (Kanaan et al., 2006; Xue et al., 2017; Hunyor and Cook, 2018) or kept under normoxia/normocapnia throughout as controls. For a broad characterization of the cardiac gene expression responses, we extracted total RNA from the LV and applied paired-end RNA-seq as a genome-wide discovery approach. Approximately 38–50 million total reads were obtained per sample with over 94% of the reads uniquely mapped to the mouse reference genome (UCSC mm9) (Supplementary Table 1).

### Microbiota Impacts Cardiac Gene Expression Under Normoxic Conditions

**FIGURE 2:**
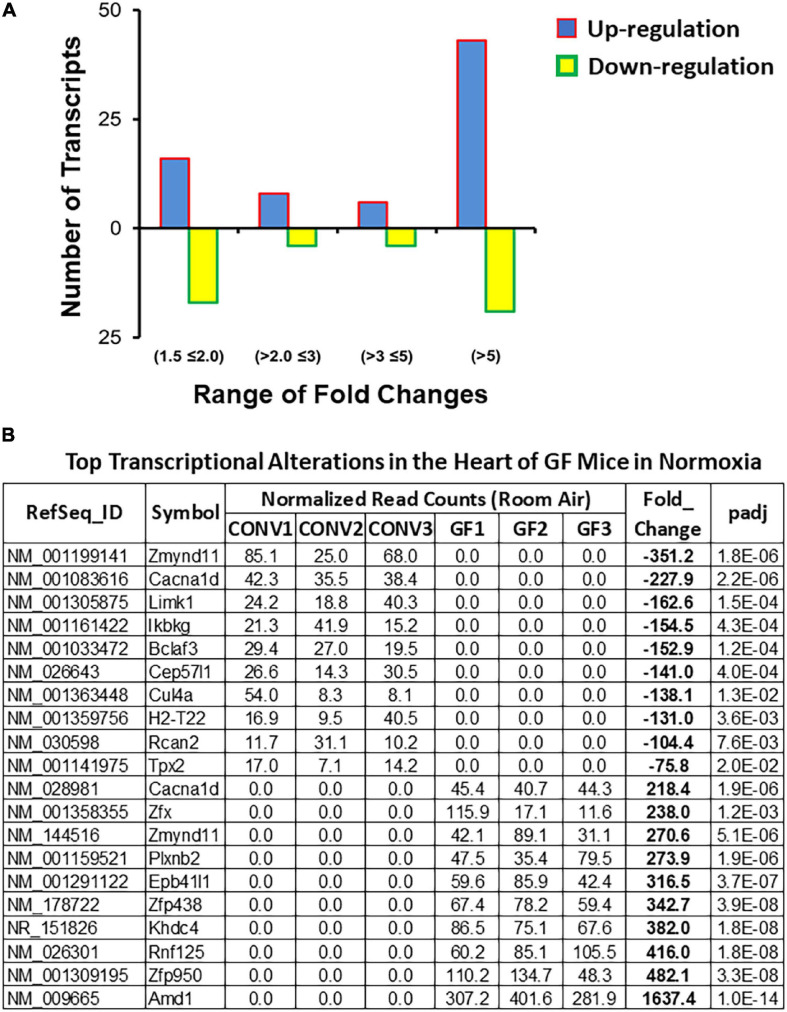
Summary of transcriptome alterations in the heart of GF mice under normoxic condition. **(A)** Distribution of fold change of the 117 differentially expressed genes (DEGs) (| Fold change| > 1.5 and Benjamini–Hochberg adjusted *p* < 0.05) in the left ventricle of GF mice as compared to CONV controls (Supplementary Table 2). **(B)** List of top 10 significantly up- or downregulated DEGs.

**FIGURE 3:**
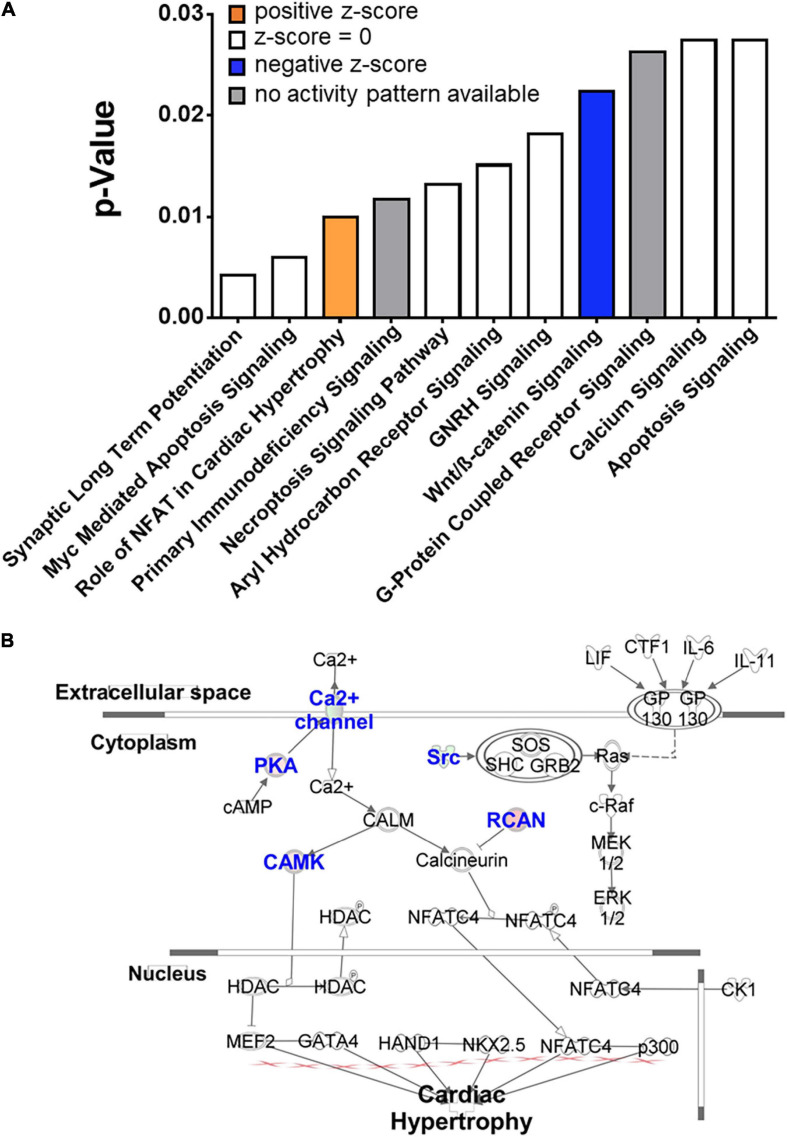
Top significantly altered cellular signaling pathways in the heart of GF mice in normoxia. **(A)** Transcriptome profiling suggested significant alterations of multiple cellular signaling pathways in the heart of GF mice (right-tailed Fisher’s exact test, *p* < 0.05, Supplementary Table 3), including **(B)** activation of the pathway regulating the “role of NFAT in cardiac hypertrophy.” Blue fonts: significantly altered components of the pathway.

We first determined the impact of the microbiota on cardiac gene expression under normoxic and normocapnic conditions. A total of 117 DEGs were identified between CONV and GF mice. Of these, 73 were upregulated and 44 were downregulated (Fold change > 1.5 and adjusted *p*-value < 0.05) in the left ventricle of GF mice as compared to CONV controls (Figure 2A and Supplementary Table 2). The most upregulated transcripts included Amd1, Zfp950, Rnf125, and Khdc4, which are involved in polyamine biosynthesis, transcription regulation, ubiquitination, and subsequent proteasomal degradation of target proteins, and pre-mRNA splicing, respectively. In contrast, the most downregulated transcripts included genes encoding a transcription repressor (Zmynd11), a Calcium Voltage-Gated Channel Subunit (Cacna1d), and a serine/threonine kinase (Limk1) (Figure 2B). In addition, several functional implications were suggested by data mining and bioinformatic analyses using the DAVID and IPA tools. Results derived from DAVID functional annotation suggested alterations in transcription regulation, alpha-actinin binding, and sarcolemma organization, as well as pathways that regulate cardiac function and activity, including the GnRH pathway, the MAPK signaling pathway, the Oxytocin signaling pathway, and vascular muscle contraction (Supplementary Table 3A). In addition, Ingenuity analysis showed potential changes in several canonical pathways that are important in cardiac function, such as the mechanisms regulating synaptic long-term potentiation, Myc-mediated apoptosis signaling, and the role of NFAT in cardiac hypertrophy (Figure 3 and Supplementary Table 3B). The different alterations on the “Myc Mediated Apoptosis Signaling” and the “Role of NFAT in Cardiac Hypertrophy” suggested that cell death and proliferation signaling pathways were differently configured in the GF mice (Supplementary Table 3B). Since (1) Myc-mediated apoptosis plays a critical role in response to stress in the myocardium (Hou et al., 2016; Veeraveedu et al., 2017; Guan et al., 2019), (2) NFAT plays a major role in cardiac hypertrophy [for selected reviews see Clerk et al. (2007), Rohini et al. (2010), Nakayama et al. (2013)], and (3) previous studies also shown that gut microbiome regulates the activity of Myc in mouse liver (Kolodziejczyk et al., 2020) and NFAT in colorectal cancer in humans (Peuker et al., 2016), our current findings suggest that alterations of Myc and NFAT signaling in GF mice may modify cardiac activity and stress response.

Furthermore, we found that 36% of the DEGs (42 out of 117) in GF mice encode nuclear proteins, including transcription factors (e.g., Dach1 and Gtf2ird2), protein kinases (e.g., Clk1 and Limk1), DNA, and histone modification enzymes (e.g., Dnmt3b and Kmt5b). Such a significant enrichment of nuclear proteins suggested differences of transcriptional regulation mechanisms between GF and CONV mice, which may lead to distinct transcriptional responses to stress in the heart.

### Distinct Transcriptional Alterations in Different Organs of GF Mice

**FIGURE 4:**
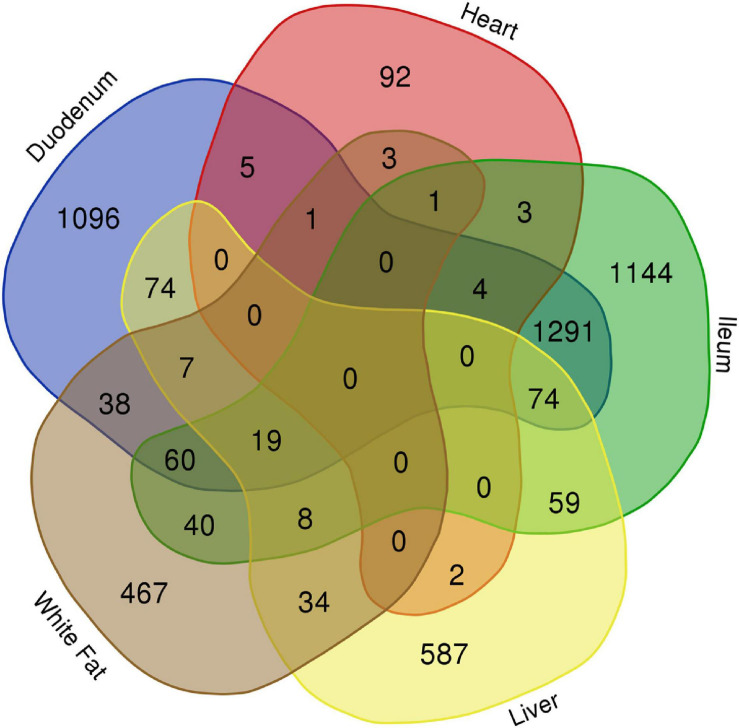
Distinct transcriptional alterations in different organs of GF mice in normoxia. Little common changes were identified in the differentially expressed genes (DEGs) between the examined organs of the GF mice under normoxic condition, except a significant overlapping between the two gut segments (duodenum and ileum), demonstrating a distinct and organ-specific impact of microbiome on gene expression.

Alterations in gene expression in GF mice have been reported in several organs (Weger et al., 2019; Mills et al., 2020). In order to determine common and distinct transcriptional responses between different organs in GF mice, we compared the reported changes (GSE77221, GEO, NCBI) with those in our study. As shown in Figure 4, each organ displayed a distinct transcriptional profile. Among the total 5,109 significantly altered transcripts (|Fold Change| > 1.5 and FDR < 0.05) in the heart, liver, white adipose tissue, duodenum, and ileum of GF mice, 3,386 (66%) were tissue specific. The largest number of unique changes was observed in the ileum (1,144 genes), followed by duodenum (1,096 genes), liver (587 genes), white fat (467 genes), and heart (92 genes), possibly reflecting the relative vicinity of the respective organs to the abundant gut microbiota. No genes were identified that were altered in all five organs, indicating a lack of common tissue responses to the microbiota. Although the vast majority of cardiac genes with significant expression changes were unique to the heart under GF conditions (79%), the most overlap was seen with the duodenum (5 genes, 4.3% of total DEGs in the heart) and the least with duodenum (or ileum) and white fat (1 gene each, <1% of total DEGs in the heart). Interestingly, the gene *cytochrome b-245, beta chain (Cybb*) was commonly altered in heart and gut, suggesting that the lack of a microbiome alters the activity of immune cells, especially neutrophils, which may lead to immune dysregulation in these organs.

### GF Condition Alters Transcriptional Responses to IHH in the Heart

**FIGURE 5:**
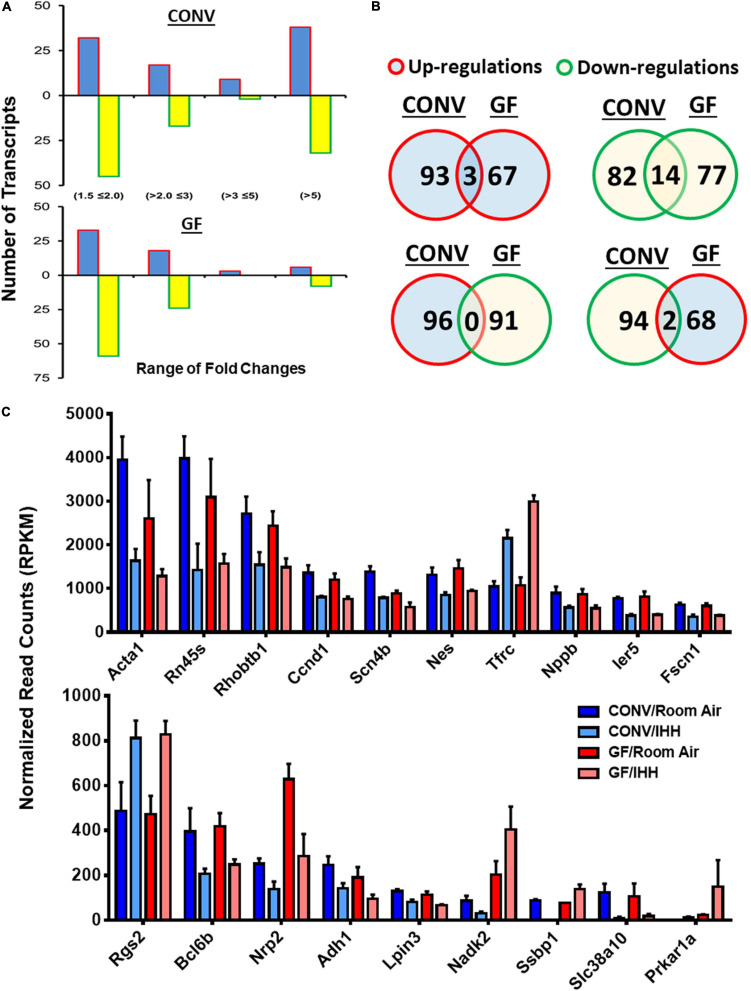
Summary of IHH-induced differentially expressed genes (DEGs) in the hearts of CONV and GF mice. **(A)** Distribution of DEGs in GF and CONV mice following IHH exposure. Note that a significantly larger proportion of the alterations >5-fold was detected in the CONV mice as compared to that in the GF mice (*p* < 0.05, chi-square test). Blue bar: up-regulations; yellow bar: down-regulations. **(B)** IHH exposure-induced common transcriptional alteration between GF and CONV mice, where 3 DEGs were upregulated and 14 DEGs were downregulated in both CONV and GF mice. Two other DEGs were downregulated in CONV but upregulated in GF mice. Red circles with blue fill-in: upregulations; green circles with yellow fill-in: downregulations. Overlaps: number of common changes observed in both CONV and GF mice. **(C)** Transcriptional abundance of the common DEGs in GF and CONV mice in room air or under IHH condition (| Fold change| > 1.5, Benjamini–Hochberg adjusted *p* < 0.05).

Previous studies have shown that stress can alter the composition of the gut microbiome, and in turn, such changes in gut microbiota may regulate stress response in various organs and tissues, including the central nervous system, the digestive system, and the immune system (Lyte et al., 2020; Park et al., 2020; Rengarajan et al., 2020; Tian et al., 2020; Vuong et al., 2020; Wang et al., 2020; Xu et al., 2020). The current study provides evidence that the microbiome may also affect stress responses in the heart through regulating transcription activities. Indeed, following 2 weeks of IHH exposure, we detected 192 significant changes in CONV mice (96 upregulated and 96 downregulated; | Fold Change| > 1.5 and FDR < 0.05) (Figure 5A and Supplementary Table 4) and 161 significant changes (70 upregulated and 91 downregulated; | Fold Change| > 1.5 and FDR < 0.05) in GF mice (Figure 5B and Supplementary Table 5). As shown in Table 1, the top DEGs in CONV mice include Rnf44, Cyb561a3, Cep250, Asph, Gm14296, and Epb41l1, whereas Kmt5a, Amd1, Tnnt2, Slc44a2, Golga5, and Cpeb3 were among the top changes in GF mice. Interestingly, only 19 transcripts were altered in both CONV and GF mice, representing around 10% of the changes in CONV and GF, respectively, demonstrating that IHH treatment induced distinct and specific transcriptional responses between CONV and GF mice. The majority of these common altered transcripts were downregulated, and the top three downregulated transcripts were Acta1 (Alpha actin 1), Rn45s (45S pre-ribosomal RNA), and Rhobtb1 (Rho-related BTB domain containing 1). On the other hand, Tfrc (transferrin receptor), Rgs2 (regulator of G-protein signaling 2), and Prkar1a (protein kinase, cAMP-dependent regulatory, type I, alpha) were the top three genes that were commonly upregulated in both CONV and GF mice. In contrast, two transcripts, Nadk2 (mitochondrial NAD kinase 2) and Ssbp1 (single-stranded DNA binding protein 1), were downregulated in CONV mice but upregulated in GF mice following IHH exposure (Figure 5C). Such dramatically different transcriptional responses suggested that IHH exposure may lead to different physiological and functional alterations in the heart of CONV and GF mice.

**TABLE 1:** Top transcriptional alterations in CONV and GF mice following IHH exposure.

**RefSeq_ID**	**Symbol**	**Normalized read counts**						**Fold change**	**Padj**
		**Air-1**	**Air-2**	**Air-3**	**IHH-1**	**IHH-2**	**IHH-3**		

**CONV Mice**									

NR_027395	Rnf44	272.4	260.7	211.7	0.0	0.0	0.0	−1406.2	6.5E-15
NM_201351	Cyb561a3	158.4	127.1	150.7	0.0	0.0	0.0	−827.7	1.8E-12
NM_008383	Cep250	141.4	111.9	138.3	0.0	0.0	0.0	−740.1	6.2E-12
NM_001286664	Ssbp1	93.6	88.9	82.6	0.0	0.0	0.0	−509.8	2.8E-10
NM_001199248	Kat5	123.6	107.7	17.6	0.0	0.0	0.0	−471.9	8.8E-07
NM_001163009	Tnik	62.4	73.3	97.8	0.0	0.0	0.0	−443.1	2.6E-09
NM_001204277	Hdac7	81.2	89.8	54.1	0.0	0.0	0.0	−422.7	5.2E-09
NR_130970_1	Gm14296	71.3	48.6	79.5	0.0	0.0	0.0	−381.8	1.3E-08
NM_008488	Arhgef1	27.4	81.0	85.5	0.0	0.0	0.0	−365.4	3.4E-07
NM_008642	Mttp	61.8	46.2	74.7	0.0	0.0	0.0	−349.2	3.0E-08
NM_001347201	Gucd1	0.0	0.0	0.0	14.4	28.5	43.9	150.7	2.3E-04
NM_001012363	Slc2a9	0.0	0.0	0.0	28.6	39.1	24.6	159.8	2.7E-05
NM_001358355	Zfx	0.0	0.0	0.0	34.6	50.0	27.8	191.7	7.3E-06
NM_178722	Zfp438	0.0	0.0	0.0	38.2	42.6	55.3	234.8	6.2E-07
NM_001167886	Kmt5b	0.0	0.0	0.0	48.3	70.0	21.8	241.4	7.3E-06
NM_001284506	Plxnb2	0.0	0.0	0.0	40.1	70.8	40.4	263.5	5.6E-07
NM_001270495	Tmem254a	0.0	0.0	0.0	51.6	54.4	48.3	266.2	1.3E-07
NM_001291122	Epb41l1	0.0	0.0	0.0	100.8	47.7	33.0	314.3	8.4E-07
NR_130970	Gm14296	0.0	0.0	0.0	67.2	61.0	85.7	367.4	8.8E-09
NM_001290367	Asph	0.0	0.0	0.0	168.1	41.5	83.9	502.3	3.9E-08
**GF Mice**									
NM_001310725	Kmt5a	332.6	616.9	544.7	0.0	0.0	0.0	−2582.6	1.9E-17
NM_009665	Amd1	321.6	421.3	295.8	0.0	0.0	0.0	−1800.0	3.6E-16
NM_001130180	Tnnt2	0.0	69299.9	70470.5	61.6	13.2	12.4	−1602.1	2.1E-03
NM_134125	Trip10	92.3	146.7	489.2	0.0	0.0	0.0	−1251.8	1.0E-07
NM_001102438	Acbd5	107.8	132.9	183.7	0.0	0.0	0.0	−734.7	1.1E-11
NM_001360080	Herc2	151.3	143.0	85.8	0.0	0.0	0.0	−657.8	4.6E-11
NM_001329143	Adamts10	94.8	120.1	117.7	0.0	0.0	0.0	−571.0	4.8E-11
NM_001361696	Slc38a10	170.4	58.5	88.2	10.3	29.6	12.4	−6.1	9.7E-04
NM_001039087	Rapgef1	767.1	518.2	501.2	156.1	160.6	283.6	−3.0	2.4E-06
NM_001243837	C7	290.4	258.7	163.4	80.0	95.4	69.2	−2.9	3.2E-06
NM_011638	Tfrc	1034.7	887.6	1268.0	2834.2	3037.6	3108.3	2.8	1.7E-28
NM_001304810	Kcnj3	37.4	11.6	29.9	79.4	115.0	66.8	3.3	1.7E-02
NM_001277328	Sox6	57.1	31.0	27.7	163.1	117.7	123.1	3.5	3.5E-05
NM_001357298	Tfrc	11.3	65.4	25.6	136.5	133.8	147.1	4.1	1.3E-02
NM_001313974	Prkar1a	26.0	24.4	23.2	256.7	24.7	170.0	6.1	1.1E-02
NM_001355067	Ctnnd1	0.0	0.0	0.0	145.9	152.4	18.6	600.8	1.7E-07
NM_001199308	Gm14440	0.0	0.0	0.0	133.6	156.4	63.1	670.1	2.0E-10
NM_198300	Cpeb3	0.0	0.0	0.0	185.3	184.8	41.5	785.4	1.5E-09
NM_013747	Golga5	0.0	0.0	0.0	151.5	128.8	200.1	912.2	9.2E-13
NM_001199186	Slc44a2	0.0	0.0	0.0	222.6	265.2	248.5	1400.1	3.3E-15

### Distinct Alterations in Cell Signaling in Response to IHH Exposure Between CONV and GF Mice

**FIGURE 6:**
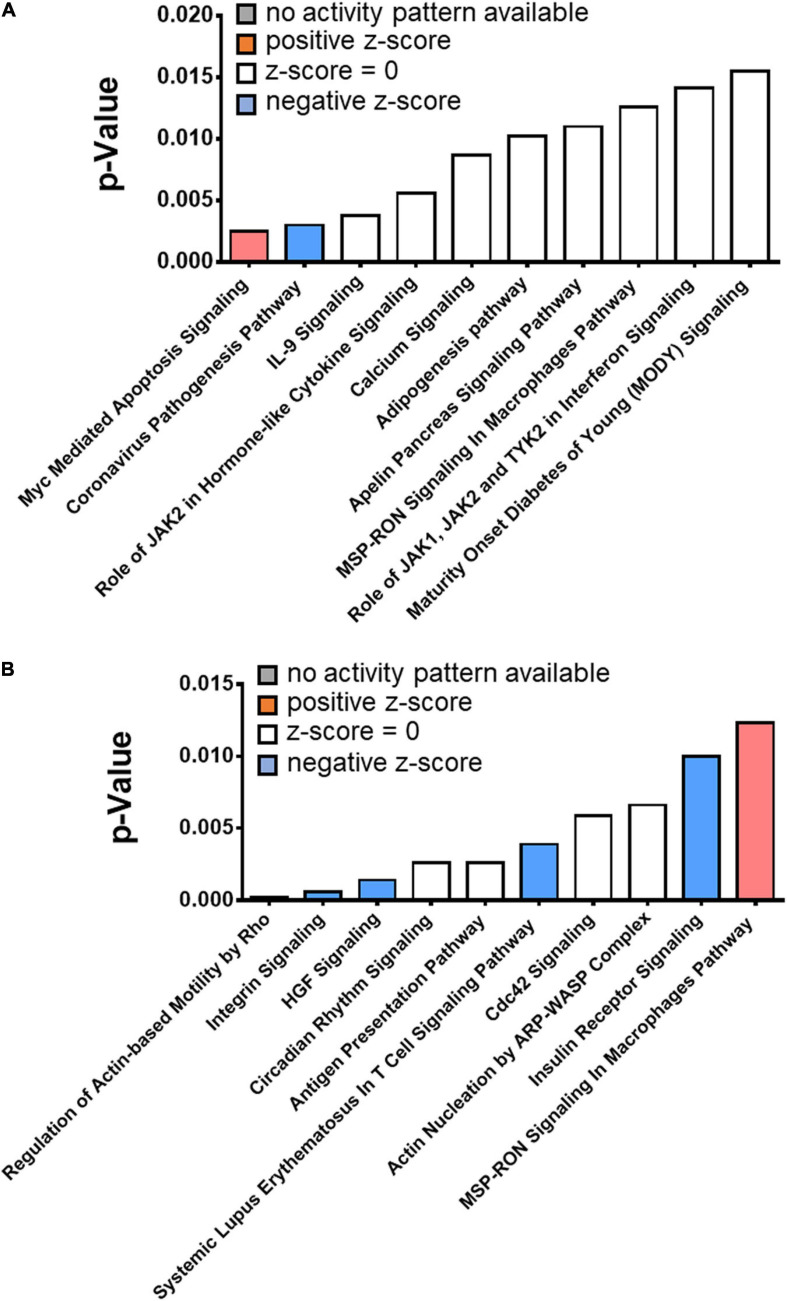
Top significantly altered pathways in the heart of CONV and GF mice following intermittent hypoxia/hypercapnia exposure. The canonical pathways that were significantly altered by IHH exposure in CONV and GF mice were determined with IPA. **(A)** The top 10 significantly altered pathways in CONV mice. **(B)** The top 10 significantly altered pathways in GF mice. The statistical significance was calculated with right-tailed Fisher’s exact test; a *p*-value < 0.05 was considered significant.

We identified distinct cell signaling mechanisms in the hearts of CONV and GF mice in response to IHH. In the CONV mice, the top significantly altered transcripts encoding a group of nuclear proteins [66/192 (34%), FDR < 0.05] that are involved in “DNA-templated transcription” [GO:0006351, 42/192 (22%), FDR < 0.05] and “regulation of DNA-templated transcription” [GO:0006355, 46/192 (24%), FDR < 0.05] (Supplementary Table 6A). The top altered signaling mechanisms were Myc mediated apoptosis signaling, coronavirus pathogenesis pathway, and IL-9 signaling (Figure 6A and Supplementary Table 6B). In GF mice, on the other hand, the significantly altered genes were involved in the biological processes that regulate cell cycle (GO:0051726, including some cyclins, e.g., Cdkn1a, Ccnd1, and Cables1, FDR < 0.05), biological rhythms (FDR < 0.05), and MHC class II protein complex (GO:0042613, FDR < 0.05) (Supplementary Table 7A). The top representative pathways were “regulation of actin-based motility by Rho,” “integrin signaling,” “HGF signaling,” and “circadian rhythm signaling” (Figure 6B and Supplementary Table 7B).

**FIGURE 7:**
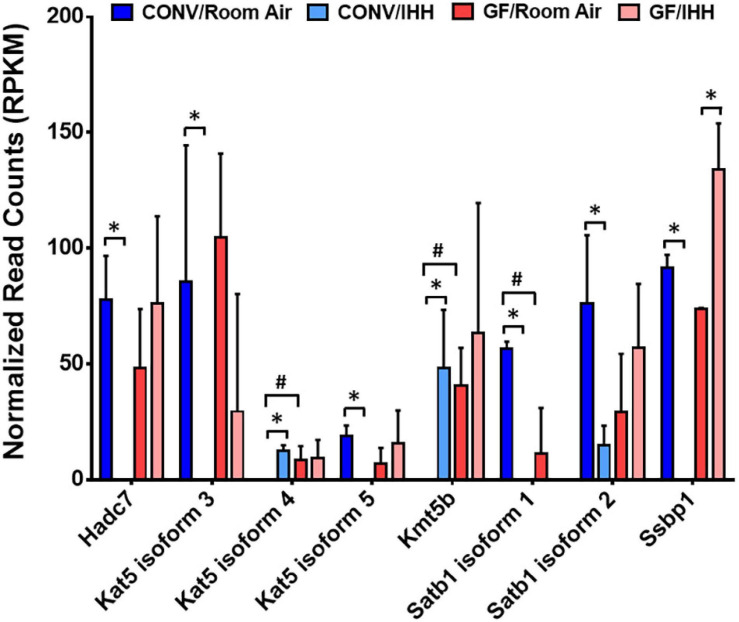
Intermittent hypoxia/hypercapnia exposure-induced changes of epigenomic modifiers in the heart of CONV mice. The expressional abundance of the differentially expressed genes (DEGs) was presented as mean ± SD for each condition. *: DEGs in CONV mice under IHH (| Fold Change| > 1.5 and Benjamini–Hochberg adjusted *p* < 0.05). #: DEGs between GF and CONV mice under room air condition (| Fold Change| > 1.5, Benjamini–Hochberg adjusted *p* < 0.05).

Following IHH exposure, “regulation of DNA-templated transcription” was one of the most significantly altered biological processes represented by 24% of the significantly altered transcripts in CONV mice. These results suggested a fine-tuning of the transcriptome, at least in part, through epigenetic modifications and rebalance of transcription regulators. Indeed, as shown in Figure 7, transcripts encoding several epigenetic modifiers were significantly changed in the CONV mice, but not GF mice, following IHH treatment. In contrast, in the heart of the GF mouse, IHH-induced transcriptional alterations suggested an array of functional/activity adjustments, such as the circadian rhythm and cell cycle genes (e.g., Per2, Clock, Cdkn1a, and Stat3) that regulate the heart rate (Yamamura et al., 2010) or the metabolism, hypertrophy, and apoptosis of cardiac cells (Bonney et al., 2013; Hauck et al., 2016; Harhous et al., 2019).

### Transcriptomic Indications of Potential Cardiac Disorders in GF and CONV Mice Following IHH Treatment

**FIGURE 8:**
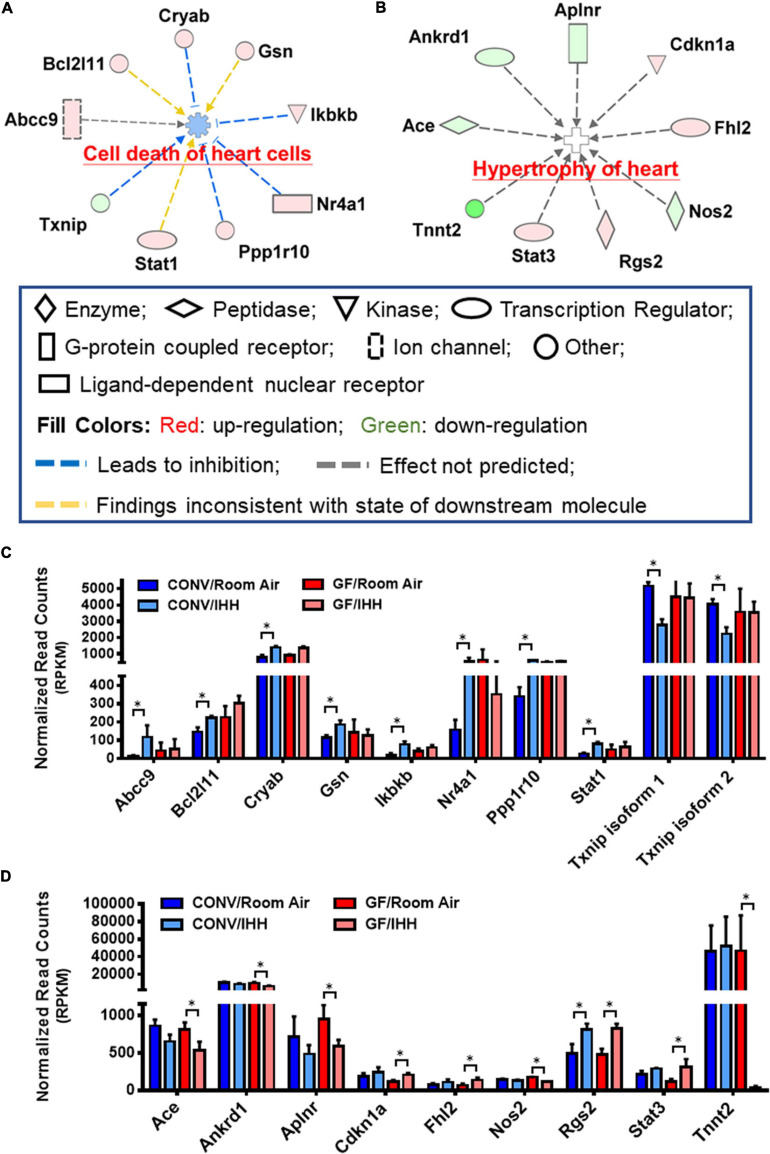
Different set of cardiovascular diseases-related genes was altered in the heart of CONV and GF mice following IHH exposure. Prediction of clinical pathology endpoints driven by IHH-induced differentially expressed genes (DEGs) suggested a significant alteration of cardiac cell death in CONV **(A)** and cardiac hypertrophy in GF mice **(B)** under IHH condition (right-tailed Fisher’s exact test, *p*-value < 0.05). **(C,D)** Expressional abundance of the related DEGs in CONV and GF mice, respectively (* | Fold Change| > 1.5, Benjamini–Hochberg adjusted *p* < 0.05).

Substantial evidence has been reported that OSA significantly increases the risk of stroke, coronary heart disease, and heart failure in humans (McEvoy et al., 2016; Drager and Lee, 2018; Benjamin et al., 2019). Interestingly, we found that the IHH-induced transcriptional alterations in the GF and CONV mice suggested distinct risks of cardiac disorders, i.e., IHH exposure evoked different sets of well-characterized pathogenetic genes in GF and CONV mice. As shown in Table 2 and Figure 8, in CONV mice, IHH-induced transcriptional alterations mainly affect cardiac necrosis and cell death. In contrast, in the GF mice, the DEGs included several key regulators of cardiac hypertrophy. In the CONV mice, changes of both well-characterized cell death stimulators and inhibitors were detected, which include BCL2-like 1 (Bcl2l1), Crystallin alpha B (Cryab), Gelsolin (Gsn), Inhibitor of nuclear factor kappa B kinase subunit beta (Ikbkb), Nuclear receptor subfamily 4 group A member 1 (Nr4a1), Protein phosphatase 1 regulatory subunit 10 (Ppp1r10), and Signal transducer and activator of transcription 1 (Stat1), as well as the downregulation of Thioredoxin interacting protein (Txnip) [for selected reviews see Wagner and Siddiqui (2009), Li et al. (2012), Ganguly et al. (2014), Wang and Yoshioka (2017), Opferman and Kothari (2018), Herring et al. (2019), Tang et al. (2019)]. In the GF mice, however, most of the disease-related DEGs were cardiac hypertrophy regulators, such as the downregulation of Angiotensin I converting enzyme (Ace), Ankyrin repeat domain 1 (Ankrd1), Apelin receptor (Aplnr), Nitric oxide synthase 2 (Nos2), and Cardiac type Troponin T2 (Tnnt2), as well as the upregulation of Cyclin-dependent kinase inhibitor 1A (Cdkn1a), Four and a half LIM domains 2 (Fhl2), Regulator of G protein signaling 2 (Rgs2), and Signal transducer and activator of transcription 3 (Stat3) [for selected reviews, see Brooks et al. (1998), Drexler (1999), Takeda et al. (2005), Tsang et al. (2010), Haghikia et al. (2011), Liang et al. (2018), Yotti et al. (2019)]. Since several genes in this group are key regulators of hypertension (e.g., Ace, Nos2, and Rgs2) (Li and Forstermann, 2000; Prichard et al., 2001; Tsang et al., 2010), it is reasonable to hypothesize that dysregulation of blood pressure might be an important mechanism regulating IHH-induced hypertrophy in GF mice.

**TABLE 2:** Transcriptome indication of potential IHH-induced cardiac disorders in GF and CONV mice.

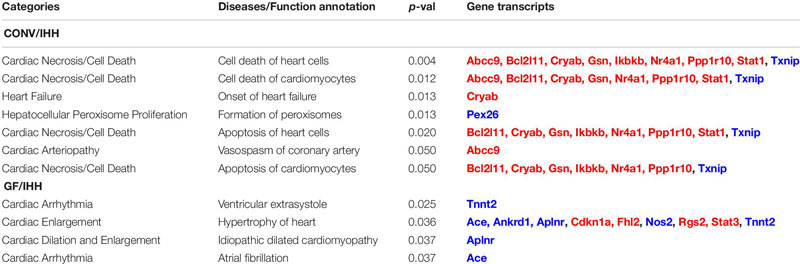

*Red font: Upregulation. Blue font: Downregulation.*

In summary, our study demonstrated that the microbiome regulates the cardiac transcriptome at baseline and in response to IHH stress. A total of 117 DEGs were identified in the heart of GF mice under normoxic condition. These DEFs encode proteins involved in transcriptional regulation, alpha-actinin binding, and sarcolemma organization. Lack of microbiome also altered cardiac transcriptional responses to IHH stress, since we found 192 IHH-induced DEGs in CONV mice and 161 DEGs in GF mice with little overlap, suggesting that IHH exposure leads to different cardiac responses between CONV and GF mice. Indeed, 24% of the DEGs detected in CONV mice are involved in the “regulation of DNA-templated transcription,” while in GF mice, IHH-induced cardiac DEGs are mostly involved in functional or activity adjustments, such as circadian rhythm and cell cycle. Future functional studies will be needed to define the functional implications of the observed DEGs. For example, IHH-induced DEGs suggest distinct risks of cardiac disorders in GF and CONV mice, since disease-related DEGs mainly affected cardiac necrosis and cell death in CONV mice, while regulators of cardiac hypertrophy were mostly observed in GF mice. Additional studies are also warranted to explore the function of specific microbial species and their metabolites on regulating cardiac gene expression under baseline and stress conditions. Gnotobiotic mouse models are uniquely suited to unravel such microbial contributions to specific physiologic responses.

## Conclusion

Even though it has been shown that OSA or IHH exposure may lead to the development of various types of cardiac disorders [for selected reviews, see Baguet et al. (2012), Dumitrascu et al. (2013), Levy et al. (2015), Turnbull (2018)], any contribution of the microbiome to this process is still largely uncharacterized. In the present study, we found that the microbiome plays an important role in the transcriptional responses to IHH stress and identified distinct responsive mechanisms in the heart of GF and CONV mice, suggesting that the microbiome modulates the cardiac function during stressful and disease conditions like OSA. Our study provides insight into the unique and common transcriptional response to IHH that is related to microbial colonization in mice. These transcriptional alterations suggest changes in cellular signaling and interaction networks regulating cardiac function, such as cardiac hypertrophy and cell death. Overall, our study is a step toward a better understanding of the role of microbiota in regulating cardiac health and disease.

## Data Availability Statement

The datasets presented in this study can be found in online repositories. The names of the repository/repositories and accession number(s) can be found in the article/Supplementary Material.

## Ethics Statement

The animal study was reviewed and approved by the Animal Care Committee of the University of California, San Diego.

## Author Contributions

DZ, JX, LE, and GH conceptualized the study, designed the experiments, and wrote the manuscript. DZ, JX, YM, and OP performed the experiments. DZ and JX performed data curation and analysis. LE and GH acquired funding and facilitated access to resources for the study. GH supervised the study. All authors contributed to the article and approved the submitted version.

## Conflict of Interest

The authors declare that the research was conducted in the absence of any commercial or financial relationships that could be construed as a potential conflict of interest.

## Footnotes

1 https://www.ebi.ac.uk/gwas/

2 https://david.ncifcrf.gov/

3 https://www.genome.jp/kegg/

4 https://reactome.org/

## Abbreviations

OSA: obstructive sleep apnea
IHH: intermittent hypoxia and hypercapnia
GF: germ-free
CONV: conventional
LV: left ventricle
HEPA: high-efficiency particulate air
DAVID: Database for Annotation Visualization and Integrated Discovery
IPA: Ingenuity Pathway Analysis
KEGG: Kyoto Encyclopedia of Genes and Genomes.
